# Numerical Inductance Calculations Based on First Principles

**DOI:** 10.1371/journal.pone.0111643

**Published:** 2014-11-17

**Authors:** Lisa F. Shatz, Craig W. Christensen

**Affiliations:** Suffolk University/Electrical Engineering, Suffolk University, Boston, MA, United States of America; University of Missouri, United States of America

## Abstract

A method of calculating inductances based on first principles is presented, which has the advantage over the more popular simulators in that fundamental formulas are explicitly used so that a deeper understanding of the inductance calculation is obtained with no need for explicit discretization of the inductor. It also has the advantage over the traditional method of formulas or table lookups in that it can be used for a wider range of configurations. It relies on the use of fast computers with a sophisticated mathematical computing language such as Mathematica to perform the required integration numerically so that the researcher can focus on the physics of the inductance calculation and not on the numerical integration.

## Introduction

Inductors are used to provide filtering or energy storage within many types of electrical systems. They are often coiled conductors whose time changing currents induce voltages either in the conductor itself or nearby conductors. They are used extensively in analog circuitry and are an essential component of every electric utility power grid. For most of the twentieth century, calculating inductances was done mainly through the use of formulas or look-up tables [1], [2] because the inductance calculation which contains an integrable singularity had been intractable. For the past thirty years or so, simulation tools such as FastHenry [3], [4], and Ansys Maxwell [5], have been used to calculate inductances. However, while FastHenty is open-sourced, the cost of Ansys Maxwell or other tools may be beyond the user's means. Also, for these tools, the user is unlikely to be familiar with their algorithms, so the researcher is less likely to develop an understanding of the fundamentals of inductance calculations; moreover an additional discretization of the inductor may be needed. Now, with the use of modern high speed computers that use sophisticated mathematical computing languages, the numerical integration is tractable and so inductances can now be computed using first principles, using a multi-functional computational program that may already be available to the user. With this method, the workings of the numerical integration are performed by the computational program, and so there is no need for the user to become an expert in sophisticated numerical integration techniques or in methods for discretizing the geometry. This method is particularly useful for the design of air core reactors, which protect equipment from potentially damaging power transients. Air reactors, which can consist of tens or even hundreds of interlaced coils, are often custom-designed for a particular operating environment, and inductance simulations of these reactors can be challenging for simulators that were designed with VLSI integrated circuits in mind. Therefore, we studied a method that uses the computational program Mathematica [6] to determine inductances.

## Materials and Methods

A formula for the self-inductance of a conductor is derived by computing the magnetic stored energy using the magnetic vector potential [7] which yields,
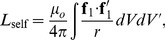
(1)where 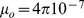 H/m; the primed variables represent the current source points and the unprimed variables, the current field points; the integral is performed over both sets of variables throughout the volume of the conductor; 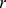 is the distance between volume elements and 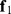 represents the ratio of the current density 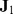 to the current 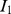, 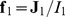. The mutual inductance 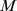 between two conductors has a similar formula except that the field points are in one conductor and the source points in another,
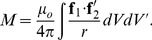
(2)


Although there are few highly symmetric configurations that allow Eqns. (1) and (2) to be integrated analytically, these equations can be integrated numerically, in theory, for any configuration of conductors, with the advent of modern computers and techniques.

### FastHenry, Ansys Maxwell, and Mathematica

FastHenry determines inductances (and resistances) by approximating a conductor as a a series of discretized rectangular filaments, each having a lumped resistance and inductance. Using mesh analysis, it derives a set of complex linear equations for the filaments (whose number may be in the thousands) and uses advanced numerical tools such as GEMRES [8], along with a multipole approach [9] to approximate the integrals of (1) and (2) that are performed for each filament. The multipole approach uses multipole expansions for (1) and (2) which may be valid for large 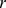 and can give an accurate approximation of the integral with far fewer computations. The user specifies the discretization by inputting the coordinates and dimensions of the rectangular filaments.

Ansys Maxwell [5] does not solve Eqns. (1) and (2) but solves Maxwell's differential equations with boundary conditions and user-specified initial conditions using a finite element method in which the solution is numerically obtained for an arbitrary geometry by breaking it down into tetrahedrons, and using second order quadratic expressions as basis functions to represent the electric or magnetic fields, at the vertices and the midpoints of selected edges. The coefficients of the basis functions are solved for using standard matrix techniques. Since Ansys Maxwell is a large finite element program for general electromagnetic problems, the overhead to use it in terms of cost and training is high.

We use Mathematica to determine the inductance by having Mathematica numerically integrate (1) or (2) over the entire volume of the conductor. Mathematica uses a global adaptive strategy, that tries to reach the required precision and accuracy goals of the integral estimate by recursive bisection of the subregion with the largest error estimate into two halves, and computes integral and error estimates for each half. The user inputs a Mathematica file which describes the integral, and does not need to perform any discretization. Mathematica determines the number of recursive bisections to determine an accurate result.

To demonstrate the accuracy of this technique, we use configurations with known inductances to check our results such as that of a ring, a rectangular loop, a solenoid, and a spiral. We used Mathematica 7 and 9 on a Dell Optiplex 2.33 GHz computer to perform the calculations. We also compare the results to those of FastHenry (FastHenry2 Porting Version 3.32), to understand the advantages and disadvantages of using this method to those of a state-of-the-art type of simulation tool. We also test our results against a simpler version of air core reactors used in the field, a two-layer, two-coiled spiral, which was built to test this method, since a known solution for this configuration does not exist.

## Results

### Inductance calculations for a ring

**Figure 1:**
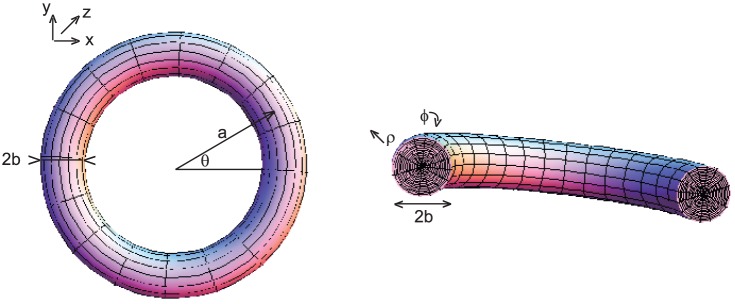
A solid ring of conductor. Left: Shown with with inner radius a-b and outer radius a+b; Right: a cross-section of the solid conductor.

A conductor in the shape of a solid ring is illustrated in Figure 1. Let us define the coordinates of a point on the conductor as,
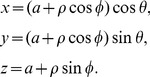
(3)


These coordinates are illustrated in Figure 1. Note that 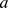 is the radius of the ring to the center of the conductor, and 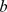 is the radius of the conductor. For a uniform current, the term 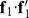 can be represented as,
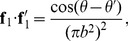
(4)and for surface current, the term 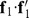 can be represented as,
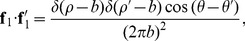
(5)where 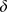 represents the Dirac delta function. The variable 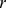 is the distance between two volume elements in the conductor, and can be defined as, 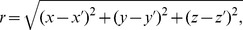
(6)which, using the formulas in Eqn. (3) becomes,
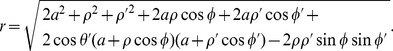
(7)


The volume element 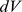 can be represented as,
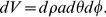
(8)


We integrate the variables, both primed and unprimed over the same range: 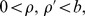 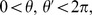 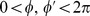. We see that there is an integrable singularity at 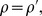 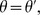 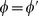. To make the numerical integration more tractable, we let 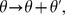 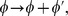 in Eqn. (7), so that the singularities are at the interval ends. Therefore, Eqns. (4)–(7) become respectively,
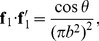
(9)

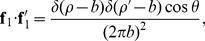
(10)

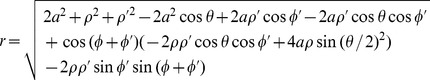
(11)


**Figure 2:**
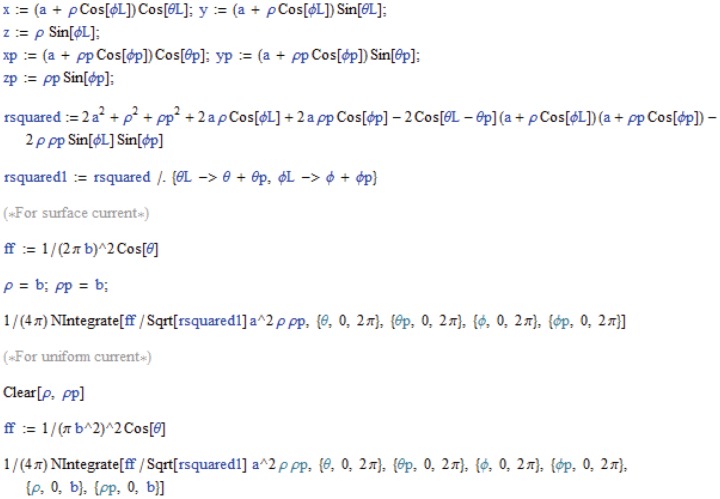
Mathematica code that performed the inductance calculations for the ring.

Substituting in Eqns. (8)–(11) into Eqns. (1) or (2) and numerically integrating yields the inductances 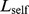 or 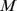. The Mathematica code for these equations is shown in Figure 2.

Because of the high degree of symmetry of the geometry of the ring, Eqn. (1) can be integrated analytically to obtain an exact solution for its inductance and we can check the numerical solution for different ring sizes against this exact solution given by,
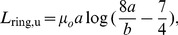
(12)for a uniform current, and by
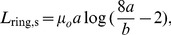
(13)for surface current [7]. The appropriateness of using the uniform current model or the surface current model can be determined by computing the skin depth 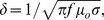 where 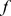 is the frequency of the current and 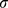 is the conductivity. Table 1 and Table 2 describe the results. If neither a surface current or a uniform current appropriately describes the current distribution, a more precise formula for the current densities can be used in Eqns. (1) or (2); however, for this configuration, and for the others described in this study, the percent differences in the results for surface and uniform current distributions are small, less than 10%, so the error in using the uniform or surface distributions for an in-between distribution would be small. The numerical integration was performed using the global adaptive method, the default integration strategy of Mathematica. The match between the exact solutions of Eqns. (12) and (13) and the results produced by this method, shown in Tables 1 and 2, is excellent with the largest error (% deviation from exact solution) being.76% but with most errors less than.01%. The small error that remained is due to the errors near the singularity, where convergence can be difficult. In fact, for almost all the inductance calculations described in this paper, Mathematica produced a warning that the global error in the numerical integration had not decreased monotonically; yet, as will be shown in this paper, all the results were highly accurate. Convergence could be obtained by lowering the precision of the result (using the PrecisionGoal option) but it was found that the results were usually not as accurate. Timing of the numerical integration was on the order of seconds for the surface current model, and tens of seconds for the uniform current model. FastHenry, which uses rectangular segments, defined by the user, produced slightly less accurate results, with the ring discretized into a 100 segments, with cross-sectional areas set to be the same as those of the rings. However, it only took seconds for both uniform currents (low frequency input) and surface currents (high frequency input), although time was needed to prepare its input files.

**Table 1:** Comparison of results between numerical method and exact solution for inductances for rings of uniform currents (Eqn. (12)).

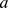 (m)	5	50	500	5000	50000	500000
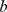 (m)	0.5	0.5	0.5	0.5	0.5	0.5
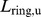 ( 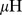 )	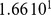	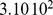	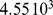	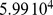	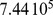	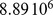
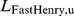 ( 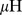 )	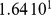	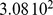	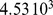	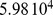	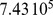	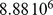
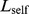 ( 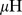 )	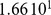	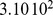	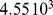	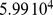	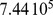	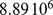
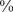 difference of 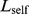 with 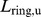	0.07	0.02	0.01	0.00	0.00	0.00
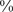 difference of 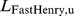 with 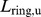	0.58	0.49	0.32	0.25	0.20	0.13

**Table 2:** Comparison of results between numerical method and exact solution for inductances for rings of surface currents (Eqn. (13)).

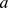 (m)	5	50	500	5000	50000	500000
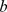 (m)	0.5	0.5	0.5	0.5	0.5	0.5
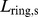 ( 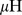 )	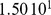	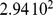	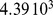	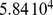	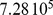	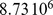
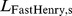 ( 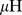 )	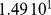	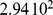	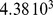	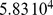	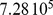	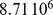
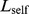 ( 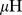 )	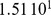	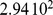	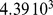	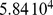	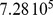	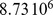
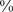 difference of 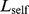 with 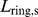	0.76	0.00	0.00	0.00	0.01	0.03
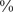 difference of 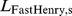 with 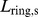	0.21	0.29	0.17	0.13	0.11	0.19

### Inductance calculations for a rectangular loop with a rectangular cross-section

**Figure 3:**
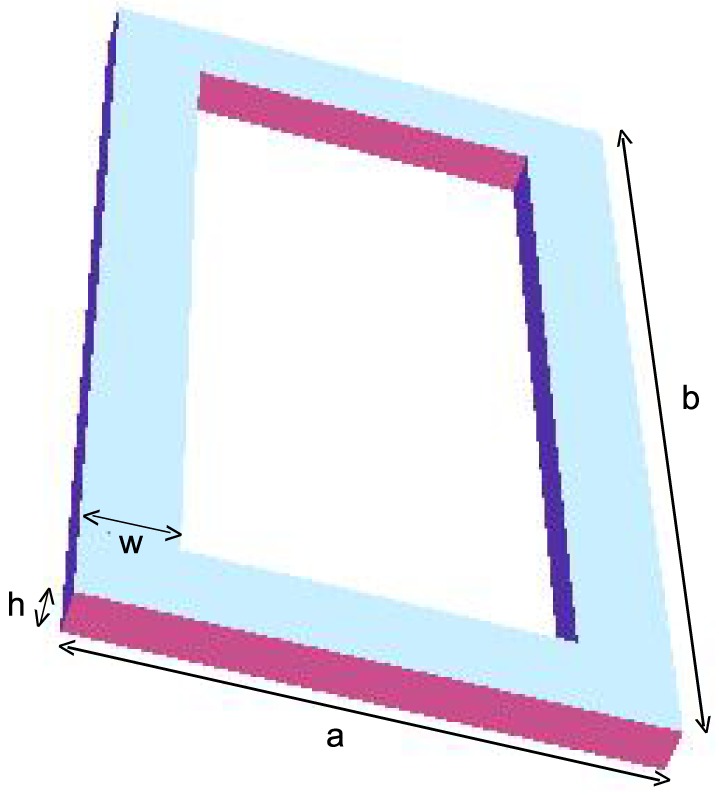
A rectangular loop.

A conductor in the shape of a rectangular loop (sides of lengths 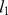 and 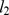) with a rectangular cross-section (height, 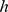, and width, 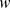) is shown in Figure 3. From symmetry, we need only compute the self-inductances of two sides with different lengths, and the two mutual inductances between opposite sides and multiply their sum by two to obtain the self-inductance of the loop. The two perpendicular sides do not add mutual inductance because the current densities are perpendicular to each other and therefore 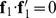. For the self-inductance of a side, for a uniformly-distributed current, 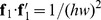, and for the mutual inductance of the opposite side, 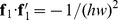. In this case, of course, we integrate in rectangular coordinates. To make the integration for the self-inductance more tractable, the numerical integration is performed with the singularities at the limits of the integration range. This geometry also has much symmetry and Eqns. (1) and (2) had been integrated analytically to obtain an exact solution [10],
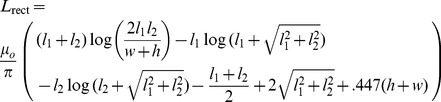
(14)


Table 3 describes the results. Again an excellent match is achieved between our numerical solution and the exact solution with the largest error being.65%. FastHenry performed slightly more accurately with its largest error being.17%. However, FastHenry was much faster, taking fractions of a second to run whereas this method took tens of seconds, which makes sense for a rectangular configuration, since FastHenry uses rectangular elements, and only four elements need to be specified as input.

**Table 3:** Comparison of results between numerical method and exact solution for inductances for a rectangular loop with a rectangular cross-section (Eqn. (14)).

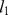 (m)	1	1	1	1
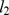 (m)	1	2	10	100
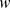 (m)	.2	.2	.2	.2
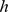 (m)	.1	.1	.1	.1
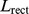 ( 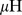 )	1.60	2.74	11.42	108.77
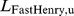 ( 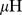 )	1.60	2.74	11.40	108.59
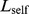 ( 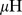 )	1.61	2.74	11.42	108.60
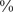 difference of 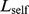 with 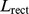	0.65	0.37	0.02	0.15
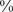 difference of 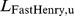 with 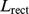	0.05	0.02	0.13	0.17

### Inductance calculations for a solenoid

**Figure 4:**
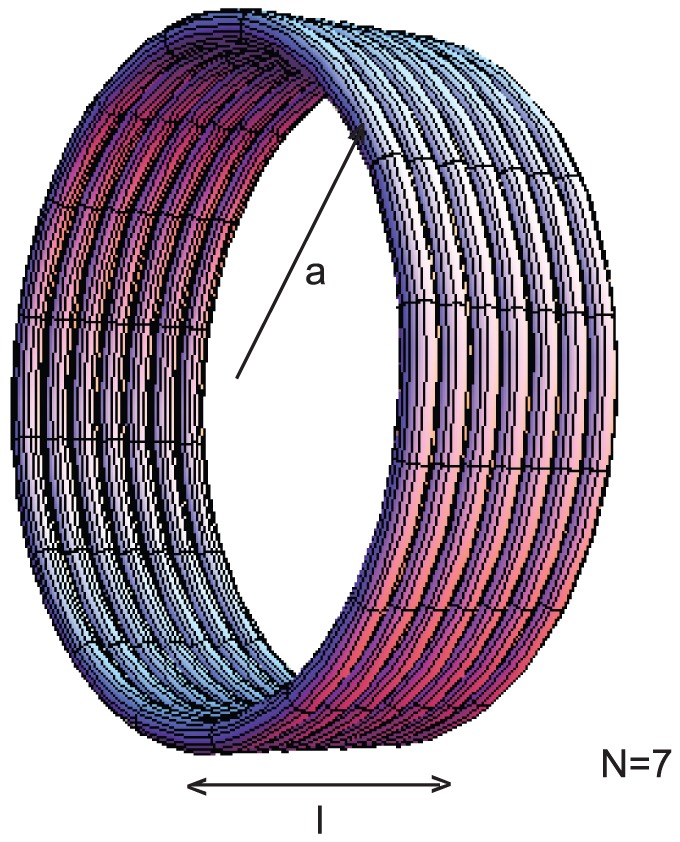
A solenoid 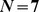 turns with nearly zero spacing between the conductors. The radius of the solenoid to the center of the conductor is 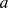, and the radius of each conductor is 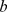. The length of the conductor is 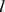, which here is equal to 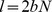 since the spacing between the conductors is nearly zero.



A conductor in the shape of a solenoid with 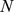 turns is illustrated in Figure 4. This calculation is nearly the same as that of the inductance of a ring except that in Eqn. (3), the 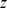 coordinate depends on the turn number 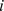,
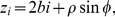
(15)where 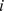 varies from 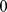 to 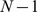, and the integration is performed over each turn. A first approximation to a solenoid is a cylinder with a surface current, whose inductance, because of its symmetry, can be obtained by analytically integrating Eqn. (1) to obtain Lorenz's exact solution 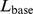 [11]. To obtain a better approximation to the solenoid inductance, 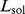 we subtract Rosa's empirically derived correction factors to account for the effect of the coils [12].

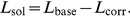
(16)

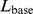
 is given by,
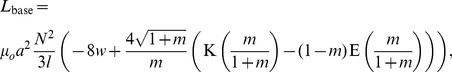
(17)where 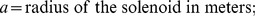 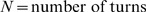 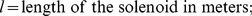 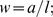 and 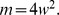 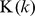 and 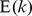 are complete elliptic integrals with 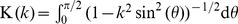 and 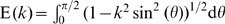. Note that there are two conventions of definitions for the elliptic integrals; Eqn. (17) used Mathematica's definition. Other conventions would use the square root of the argument.


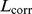
 can be written as, 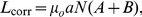
(18)where 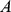 and 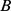 are empirically derived constants; 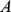 depends on the ratio of the diameter of the conductor to the turn pitch, and 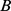 depends on 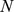. Values for 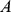 and 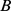 have been tabulated [13] and numerical values for all these quantities can be found online[14]. Table 4 describes the results for solenoids of various radii and number of turns. The match is again excellent with errors less than 1.4%. This method worked considerably better than FastHenry. For 5-turn solenoids, with 100 elements, its largest error was 5.1%. Increasing the number of discretization segments did not reduce the error. This method ran for 1–3 minutes and FastHenry took 2 minutes. For a 100-turn solenoids with 1000 elements, FastHenry ran out of memory and did not produce a result. While this method produced an accurate result for 100-turn solenoids, it did take 1–6 hours to run, with the smaller radius, giving the larger run-time.

**Table 4:** Comparison of results between numerical method and the Lorenz method with the Rosa corrections for inductances for solenoids with surface currents (Eqn. (16)).

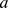 (m)	50	50	500	500	5000	5000
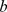 (m)	.5	.5	.5	.5	.5	.5
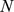	5	100	5	100	5	100
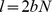 (m)	5	100	5	100	5	100
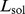 ( 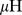 )	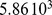	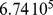	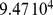	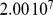	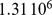	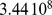
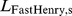 ( 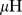 )	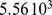	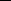	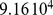	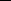	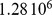	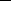
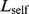 ( 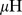 )	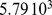	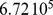	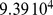	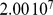	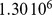	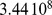
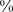 difference of 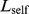 with 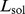	1.35	0.22	0.84	0.08	0.60	0.05
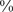 difference of 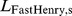 with 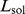	5.07	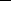	3.87	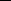	2.20	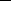

### Inductance calculations for a spiral

**Figure 5:**
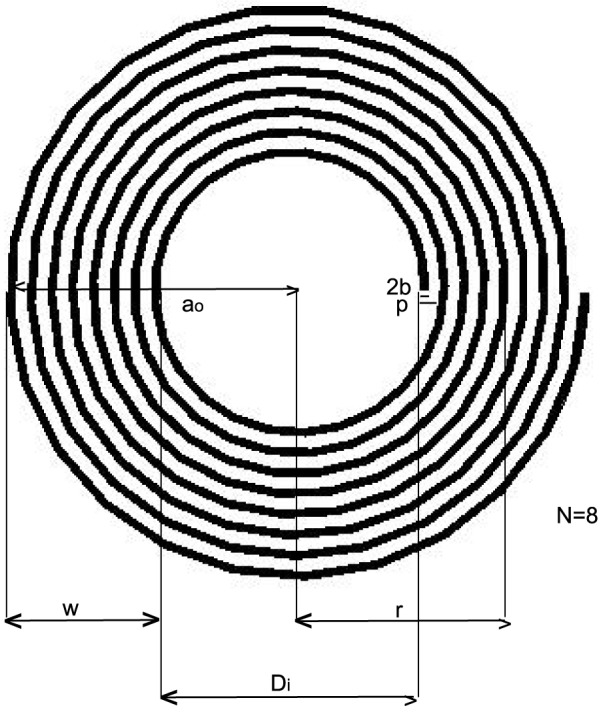
A conductor in the shape of a spiral. The radius 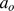 extends to (but does not include) the outermost spiral. 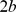 is the diameter of the conductor and 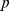 is the pitch. For Wheeler's formula for the inductance of a spiral, the parameters 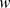, the width of the turns, and 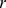 the radius to the center of the turns, are needed. 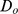 is the inner diameter which is used to calculate 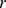.

Another check on this method is to compare 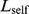 for a flat spiral (Figure 5) with that of Wheeler's empirical formula [15]. To determine 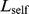, we use the coordinates described in Eqn. 3 with 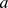, the radius of the spiraled conductor, described by,
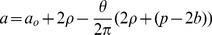
(19)where, 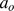 is the widest radius of the spiral (not including the outermost conductor), 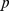 is the pitch of the turns which equals the diameter 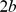 of the conductor plus the spacing between the conductors. Note, for the spiral, there is no axisymmetry and we can no longer let 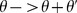 (although we continue to let 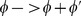); moreover, the limits of 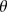 are from 0 to 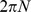. The empirical formula is given by,
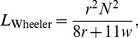
(20)where 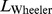 is the inductance in *µ*H, 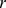 is the radius to center of the turns in inches, and 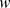 is the width of the turns, in inches. We can obtain 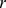 and 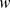 using the following formulas,
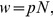
(21)

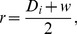
(22)where 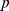 is the pitch in inches and 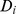 is the inner diameter of the spiral, which can be obtained from,
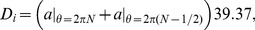
(23)where the factor 39.37 converts from meters to inches. Wheeler's formula is accurate to within 5% when there are sufficient number of turns (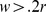), when the spacing between the turns is small, and when the skin effect is not important. As can be seen from Table 5, the results for 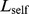 match to within 10% the results of 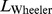 for the uniform current model, and match to within 6% for the surface current model. The match for FastHenry was considerably worse, with the largest error of 42% for a uniform current and with the largest error of 39% for surface current. Doubling the number of elements in the input file for FastHenry did not reduce the error. The run-time for this method was a few minutes and did not increase with the number of spiral turns while for FastHenry, the run-time for the 4-turn spiral, with 100 elements was 20 seconds, and the run-time for the 15-turn spiral which had 500 elements was 10 minutes.

**Table 5:** Comparison of the results of between numerical calculation and Wheeler's approximate formula 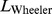 (Eqn.(20)) for a spiral (Figure 5).

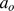 (m)	0.768	0.768	0.768	1.530	1.530
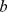 (m)	0.009	0.009	0.009	0.009	0.009
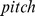 (m)	0.027	0.027	0.027	0.027	0.027
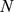	10.0	8.0	4.0	15.0	7.0
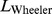 ( 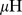 )	106.4	89.8	37.9	642.5	238.5
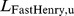 ( 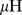 )	150.9	113.2	42.8	856.9	281.8
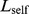 ( 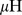 ) (uniform current)	110.1	91.0	37.8	628.3	230.3
% difference of 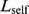 (uniform current) with 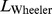	3.5	1.3	0.2	2.3	3.4
% difference of 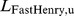 with 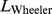	41.8	26.8	12.9	33.4	18.2
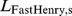 ( 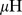 )	147.9	115.6	41.6	846.6	276.9
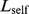 ( 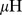 ) (surface current)	102.1	84.3	36.6	615.9	232.3
% difference of 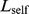 surface current) with 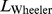	4.0	6.1	3.4	4.3	2.6
% difference of 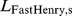 with 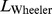	39.0	28.7	9.8	31.8	16.1

### Inductance calculations for a two-layer spiral with two coils

**Figure 6:**
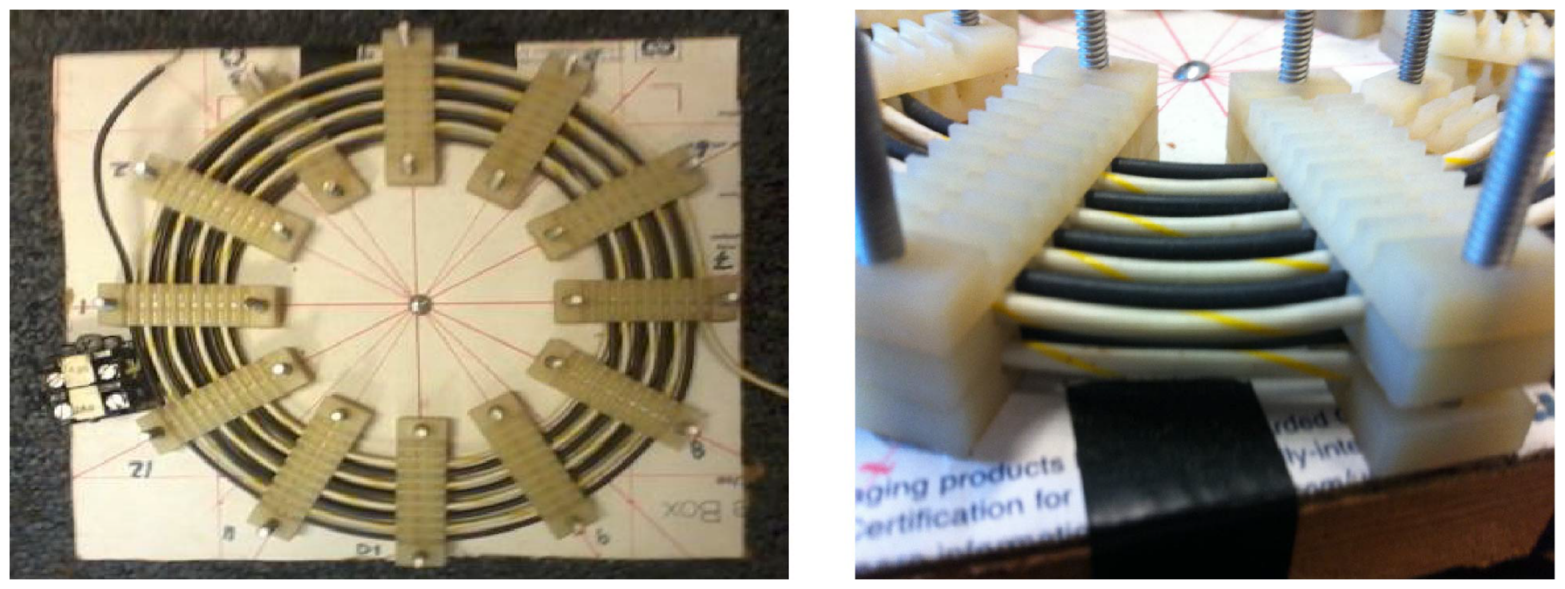
A two-layered spiral transformer with two coils, constructed to test the method presented in this paper. Left: The top view; the two spirals are interlaced and there are two layers of the them. Right: Side view. The formula for its shape is described in Eqn. (3), (15), and (19); its dimensions are given in the caption of Table 6.

Shown in Figure 6 is a two-layer spiral with two coils that was built to test this method. As can be seen from the figure, this configuration consists of two interlaced coils that are arranged in two layers of spirals. From basic circuit theory of non-ideal transformers [16] when a transformer's inductance is measured between the ends of each coil with the coils connected in parallel, its inductance is equal to,
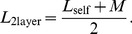
(24)


By using Eqn. (3) as applied to a single layer spiral (Eqn. (19)), with Eqn. (15) (since we have two layers) for each of the two coils, and also by computing their mutual inductance (Eqn. (2)), we can compute 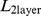. The dimensions and the inductances of both the model and the measurements are shown in Table 6. The measurements were made using an Agilent LCR meter. We see that the results match the measurements to within 2%, which is likely within the accuracy of the measurement considering stray inductance at the ends of the spiral and of the probe leads.

**Table 6:** Measurements of the two layered spiral shown in Figure 6. The dimensions are 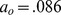
m, 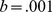
m, 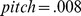
m, and 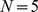, and the measured inductance is 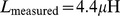.

Current Distribution	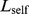 ( 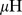 )	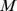 ( 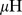 )	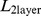 ( 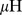 )	Error (%)
Uniform	4.55	4.06	4.30	2.2
Surface	4.60	4.38	4.49	2.0

## Discussion

From the results for inductors for which the exact solutions for their inductances are known, such as that of a ring, we see that numerically integrating the inductance formula derived from energy formulations is highly accurate, and likely more accurate than previously used empirical methods that do not claim to give a highly accurate estimate. Moreover, this method, in theory, can be used for any configuration of inductors. The disadvantages of this method are that the length of time of the numerical integration may be long, if the program is run on a slow computer, and errors may be introduced because of the difficulty of convergence near the singularity. We performed the numerical integration inside of Mathematica which meant that it ran on a virtual machine, increasing its run-time, instead of directly on the CPU. Running the code in C directly on the CPU would likely eliminate any disadvantage of long run-times.

### Limitations of the study, open questions, and future work

This study showed that inductance calculations based on first principles can be performed on several different coil formations using a sophisticated mathematical computing language which performed the numerical integration of the singularity so that the researcher does not need to become expert in numerical integration techniques or expert in how a simulator such as FastHenry or Ansys Maxwell works. However, the researcher still needs be cognizant of the underlying principles of the inductance calculation which may be important in sharpening his skills in inductor design. Note that this study was limited to only five types of inductor configurations; it stands to reason that this method can be applied to other configurations although this needs to be proved. Another caveat is that almost always the global error of the numerical integration did not decreased monotonically, although accurate results were obtained nonetheless. An additional drawback of this method is that the run-time becomes large when simulating more complex configurations. However, FastHenry did not produce a result for these complex configurations although it is suspected that a greater understanding of its workings may allow the user to fix the problem. Future work might entail performing the numerical integration outside of the Mathematica environment so that the calculation runs directly on the CPU and therefore the runtime would be decreased.

## Conclusion

A versatile method based on first principals of computing the inductances of conductors has been described and shown to be accurate, with its accuracy limited by the technique used to numerically integrate the inductance formula. This method allows for the calculation of an inductance using the precise geometry of the inductor particularly an air reactor, or transformer, whose inductance may not have been previously derived or present in look-up tables and empirical formulas. It also eliminates the need to for the user to focus on the numerical aspects of the problem at the expense of the physics of the calculation. Since, in theory, this method will work for any geometry, it can facilitate the design of inductors and transformers to meet particular specifications. This method could not have been used before the advent of fast computers due to the computationally intense method for numerical integration; however, now it is practical and likely will be used to develop inductors and transformers whose inductances would previously haven been exceeding difficult to derive. This method proved to be more robust for calculating the inductances of certain solenoids and spirals for at least one other open-sourced simulator.
